# A Medical Student Curriculum on Functional Medical Disorders

**DOI:** 10.1111/tct.70117

**Published:** 2025-06-26

**Authors:** Mohsin F. Butt, Daniele Scotto, Christopher G. S. Gilmartin, Arkadeep Dhali, Sherif Gonem, Sumeet Singhal, Tanya M. Monaghan, John Frain, Maura Corsetti

**Affiliations:** ^1^ NIHR Nottingham Biomedical Research Centre Nottingham University Hospitals NHS Trust and the University of Nottingham Nottingham UK; ^2^ Nottingham Digestive Diseases Centre, Translational Medical Sciences, School of Medicine University of Nottingham Nottingham UK

**Keywords:** curriculum development, functional medical disorders, medical education

## Abstract

**Background:**

Functional medical disorders (FMDs) refer to persistent physical symptoms that cause impairment or disability, but which cannot be explained by routine medical testing. Negative perceptions towards FMDs exist amongst a variety of healthcare professionals, including medical students. The aim of this study was to develop and evaluate a course addressing FMDs for medical students in their first year of clinical training, which was integrated within the formal medical school curriculum.

**Approach:**

A multidisciplinary team of eight healthcare professionals delivered seven teaching sessions, each two hours in length, over six weeks. The curriculum was delivered via a combination of didactic teaching, small group tutorials and sessions with simulated patients. Pre‐ and post‐course validated questionnaires assessed knowledge of, attitudes towards and confidence around irritable bowel syndrome (IBS). Students undertook a two‐station objective structured clinical examination (OSCE), which assessed their ability to take a clinical history from and communicate a diagnosis to simulated patients with IBS and fibromyalgia.

**Evaluation:**

Twenty‐seven students completed the pre‐ and post‐course surveys, which demonstrated an increase in knowledge of FMDs at course conclusion (88.89% vs. 57.50%, *p* < 0.001). Students' confidence ratings increased in all (100%) domains relating to FMDs: pathophysiology, symptoms, investigations for diagnosis and communicating a diagnosis (*p* < 0.001, all analyses). There was a statistically significant improvement in attitude ratings towards FMDs in 11 of 12 (91.7%) questions. All (100%) students passed the OSCE.

**Implications:**

A course integrated within the formal medical school curriculum may be a helpful way to improve knowledge and reduce stigma around FMDs.

## 1 Background

Functional medical disorders (FMDs) refer to persistent and troublesome physical symptoms that result in impairment or disability [1]. Examples of FMDs include irritable bowel syndrome (IBS), fibromyalgia, non‐cardiac chest pain, functional breathlessness and functional neurological disorder. In UK settings, FMDs account for one‐in‐five consultations in primary care [2], and their prevalence is as high as 66% in secondary care [3]. FMDs are associated with numerous indirect and direct costs; at least 10% of the total UK National Healthcare Service expenditure is directed towards supporting patients who have such conditions [4].

Despite their high prevalence, a recent PRISMA systematic review uncovered a paucity of teaching activities and research around FMDs in the medical curriculum, suggesting that medical graduates may be ‘ill‐equipped’ to manage people living with such conditions [5]. The under‐representation of FMDs within the medical school curriculum may be attributed to several factors, including the complexity of teaching conditions with unclear aetiology [6], negative attitudes from tutors and clinicians towards people living with FMDs [7] and the perceived low priority of FMDs within an overloaded medical curriculum [5]. Unfortunately, lack of teaching around FMDs may propagate negative attitudes towards patients who have such conditions [8].

The primary aim of this study was to develop a curriculum addressing FMDs for students in their first year of clinical training, which was integrated within the formal medical school course. The secondary aim was to evaluate the effectiveness of the course using two methods: (i) pre‐ and post‐course surveys that assessed knowledge of, attitudes towards and confidence around FMDs and (ii) a post‐course objective structured clinical examination (OSCE). Data were presented in abstract form at the British Society of Gastroenterology Annual Conference 2024 [9].

*The primary aim of this study was to develop a curriculum addressing functional medical disorders for students in their first year of clinical training, which was integrated within the formal medical school course*.

## 2 Approach

Four healthcare professionals (a trainee gastroenterologist [M.F.B.], a primary care physician [J.F.], a consultant gastroenterologist [T.M.M.] and a consultant neurogastroenterologist [M.C.]) developed a curriculum that addressed the learning objectives in Table 1. Learning objectives were delivered through a combination of didactic teaching, small group tutorials and sessions with simulated patients. A consultant respiratory physician [S.G.] and a consultant neurologist [S.S.], both experts in managing FMDs in their respective specialties, were invited to deliver relevant sessions.

**TABLE 1:** Learning objectives.

Session title	Learning objectives
1. Introductory session	Define functional medical disorders (FMDs): Understand and explain the concept of FMDs, including their definition, characteristics and differentiation from organic medical conditions. Explain the biopsychosocial model: Describe the interplay between biological, psychological and social factors in the genesis and evolution of FMDs. Discuss epidemiology and risk factors: Analyse epidemiological data related to FMDs and identify key risk factors associated with their onset and exacerbation. Explore diagnostic challenges: Evaluate the diagnostic challenges and controversies surrounding FMDs, including the role of clinical assessment, differential diagnosis and potential comorbidities. Review treatment approaches: Examine evidence‐based treatment approaches for FMDs, including pharmacological, psychological and multidisciplinary interventions aimed at symptom management and improving quality of life.
2. Clinical communication skills	Demonstrate empathetic communication: Practice empathetic communication techniques with simulated patients presenting with symptoms consistent with FMDs, showing sensitivity to their experiences. Explore patient narratives: Effectively elicit and explore patient stories to understand the onset, progression and impact of FMDs, using open‐ended questions and active listening. Explain FMDs: Clearly explain key concepts of FMDs to simulated patients, including the biopsychosocial model and treatment options. Manage patient expectations: Skilfully manage patient expectations regarding diagnosis and treatment of FMDs, addressing misconceptions and discussing realistic outcomes. Engage in shared decision‐making: Participate in shared decision‐making processes with simulated patients regarding treatment options, considering their preferences, values and goals.
3. Irritable bowel syndrome	Define and describe irritable bowel syndrome (IBS): Define IBS and describe its clinical presentation, including common symptoms, such as abdominal pain and changes in bowel habits. Explain pathophysiology: Explain the underlying pathophysiology of IBS, including factors related to a dysregulated gut‐brain axis. Discuss diagnostic criteria: Discuss the diagnostic criteria for IBS, including the Rome IV criteria, and differentiate between IBS subtypes (constipation, diarrhoea and mixed). Explore treatment options: Explore evidence‐based treatment options for managing IBS, including dietary modifications, pharmacotherapy and psychological interventions. Address patient management strategies: Develop strategies for managing patients with IBS, including patient education, lifestyle modifications and approaches to improve patient‐provider communication and shared decision‐making.
4a. Functional dyspepsia	Define functional dyspepsia: Define functional dyspepsia and differentiate it from organic causes of dyspepsia, such as peptic ulcer disease and gastro‐oesophageal reflux disease. Describe clinical features: Describe the clinical features and common symptoms of functional dyspepsia, including epigastric pain or discomfort, early satiety and postprandial fullness. Discuss pathophysiology: Discuss the underlying pathophysiology of functional dyspepsia, including factors such as gastric motility disturbance and visceral hypersensitivity. Review diagnostic criteria: Review the diagnostic criteria for functional dyspepsia, including the Rome IV criteria, and distinguish between subtypes (epigastric pain syndrome and postprandial distress syndrome). Explore management strategies: Explore evidence‐based management strategies for functional dyspepsia, including lifestyle modifications, pharmacotherapy, dietary interventions and psychological therapies.
4b. Nausea and vomiting disorders	Understand Rome IV criteria: Gain a thorough understanding of the Rome IV diagnostic criteria for nausea and vomiting disorders, including the definitions and classification of different subtypes. Apply Rome IV criteria in clinical practice: Learn how to apply the Rome IV criteria effectively in clinical settings to diagnose and classify patients with functional nausea and vomiting disorders. Differentiate subtypes: Differentiate between the various subtypes of nausea and vomiting disorders as classified by Rome IV, such as chronic nausea, cyclical vomiting syndrome and cannabinoid hyperemesis syndrome. Discuss diagnostic challenges: Discuss the diagnostic challenges and considerations specific to using Rome IV criteria for nausea and vomiting disorders. Explore management strategies: Explore evidence‐based management strategies tailored to different subtypes of nausea and vomiting disorders classified by Rome IV, including pharmacological, dietary and behavioural interventions.
5a. Narcotic bowel syndrome	Define narcotic bowel syndrome: Define narcotic bowel syndrome and differentiate it from other disorders of gut‐brain interaction, emphasising its association with long‐term opioid use and the development of paradoxical hyperalgesia. Describe clinical features: Describe the clinical features and symptoms of narcotic bowel syndrome, including continuous abdominal pain, despite ongoing opioid use. Explain pathophysiology: Explain the underlying pathophysiological mechanisms proposed for narcotic bowel syndrome, including opioid‐induced hyperalgesia, gastrointestinal dysmotility and opioid receptor adaptation. Discuss diagnostic challenges: Discuss the challenges in diagnosing narcotic bowel syndrome, including distinguishing it from other disorders of gut‐brain interaction. Explore Management Strategies: Explore evidence‐based management strategies for narcotic bowel syndrome, including opioid tapering or cessation, multimodal pain management approaches, behavioural therapies and supportive care for symptom relief.
5b. Functional rheumatological disorders	Define functional rheumatological disorders: Define and differentiate functional rheumatological disorders, including fibromyalgia, from organic rheumatological conditions based on the absence of identifiable structural or inflammatory abnormalities. Describe fibromyalgia symptoms: Describe the characteristic symptoms of fibromyalgia, such as widespread musculoskeletal pain, fatigue, sleep disturbances and cognitive difficulties. Discuss pathophysiology of fibromyalgia: Discuss current theories on the pathophysiology of fibromyalgia, including central sensitisation, dysregulated pain processing pathways and neuroendocrine abnormalities. Explain diagnostic criteria: Explain the diagnostic criteria for fibromyalgia and discuss the challenges and controversies surrounding its diagnosis. Explore management strategies for fibromyalgia: Explore evidence‐based management strategies for fibromyalgia, encompassing pharmacological treatments, non‐pharmacological approaches (e.g., exercise and cognitive behavioural therapy) and patient education to improve symptom management and quality of life.
6. Functional neurological disorders	Define functional neurological disorders: Define functional neurological disorders and distinguish them from organic neurological conditions based on the absence of structural abnormalities and the presence of neurological symptoms that are inconsistent or incongruent with known neurological diseases. Describe clinical presentations: Describe the common clinical presentations and symptoms of functional neurological disorders, such as motor symptoms (e.g., weakness, tremor), sensory symptoms (e.g., numbness, tingling) and dissociative seizures. Discuss psychosocial factors: Discuss the role of psychosocial factors, including stress, trauma and psychological distress, in the development and exacerbation of functional neurological disorders. Explain diagnostic criteria: Explain the diagnostic criteria for functional neurological disorders, emphasising the importance of clinical evaluation, exclusion of organic causes and recognition of specific functional neurological disorder subtypes (e.g., functional movement disorders, dissociative seizures). Explore multidisciplinary management: Explore evidence‐based multidisciplinary management strategies for functional neurological disorders, including neurology input, psychological therapies (e.g., cognitive behavioural therapy), physical therapy and patient education.
7. Functional respiratory disorders	Define functional respiratory disorders: Define and distinguish functional respiratory disorders from organic respiratory conditions based on the absence of identifiable structural abnormalities and the presence of respiratory symptoms that are disproportionate to physiological findings. Describe clinical presentations: Describe the common clinical presentations and symptoms of functional respiratory disorders, such as breathlessness, chest pain, coughing and hyperventilation syndrome. Discuss psychosocial and behavioural factors: Discuss the role of psychosocial factors (e.g., anxiety, stress) and behavioural patterns (e.g., maladaptive breathing habits) in the development and exacerbation of functional respiratory disorders. Explain diagnostic challenges: Explain the challenges in diagnosing functional respiratory disorders, including the need to rule out organic causes and the reliance on clinical assessment and exclusion criteria. Explore management strategies: Explore evidence‐based management strategies for functional respiratory disorders, including education on normal respiratory physiology, breathing retraining techniques (e.g., diaphragmatic breathing), cognitive behavioural therapy and multidisciplinary care approaches.

*Note: Disorder of gut‐brain interaction* is the new accepted terminology for *functional gastrointestinal disorder*.

Abbreviations: FMD, functional medical disorder; IBS, irritable bowel syndrome.

Since clinical history taking is crucial for diagnosing FMDs, communication skills sessions were designed to train students to effectively collect clinical histories from simulated patients—lay persons or actors who adopt and adapt a patient scenario [10]. Students were taught to make a ‘positive diagnosis’ of an FMD, focussing on identifying specific characteristics of the condition rather than excluding an exhaustive list of conditions.

## 3 Evaluation

### 3.1 Assessment of Knowledge, Confidence and Attitudes

Based on the collective experience of the course convenors and disease prevalence, the assessments were primarily oriented around IBS, a prototypical FMD with a high population prevalence. Students completed pre‐ and post‐course surveys, which assessed their knowledge of IBS and confidence in understanding its pathophysiology, recognising symptoms, establishing a diagnosis, selecting appropriate investigations and communicating the diagnosis to patients (Data S1). Students completed identical confidence‐based questions around inflammatory bowel disease (IBD), an organic gastrointestinal disorder.

To assess attitudes, students read two vignettes—one addressing a patient with IBS and another with IBD (Data S1). Lastly, participants answered two questions that assessed burnout.

Survey items were adapted from a previously published non‐interventional cross‐sectional study that assessed US medical students' knowledge, attitudes and confidence towards IBS versus IBD [11].

### 3.2 Objective Structured Clinical Examination

The final mark in the course was based on students' performance in a two‐station post‐course OSCE that adhered to the standards set by the University of Nottingham, aligned with guidance from the UK Medical Schools Council. For the 2023 assessment, students were asked to take a history from and communicate a diagnosis to simulated patients with IBS and fibromyalgia.

### 3.3 General Post‐Course Feedback

Students completed a general feedback form (Data S2) at the end of the course and were offered the opportunity to have feedback recorded on a video camera after signing another consent form (Data S3).

### 3.4 Statistical Analysis

A chi‐squared test was used to assess differences in knowledge‐based (true/false) responses between pre‐ and post‐course surveys. Attitudes and confidence were measured using Likert scales, and differences in mean scores were analysed using paired sample *t*‐tests. A Spearman rank correlation coefficient ascertained the relationship between students' attitudes towards patients with IBS (calculated from the mean value of the final four attitude‐based questions: patience, optimism, resilience and honesty) and burnout (calculated from the mean value of two burnout questions). The Angoff method established the OSCE pass mark, and results were standardised to the university pass threshold.

Statistical computations were performed using JMP (SAS Institute Inc.). Categorical data were summarised as number (%); continuous data were summarised as mean (SD). *p* < 0.05 were considered significant.

### 3.5 Ethical Approval

This study was approved as an educational evaluation by the University of Nottingham and was exempt from formal research ethics review (FMHS 44‐1023).

## 4 Results

### 4.1 Demographics

Twenty‐nine third‐year medical students enrolled in the course and two‐point survey data were collected from 27/29 participants: six (22.2%) male and 21 (77.7%) female students with a mean [SD] age of 20.52 [0.7].

### 4.2 Knowledge

**FIGURE 1:**
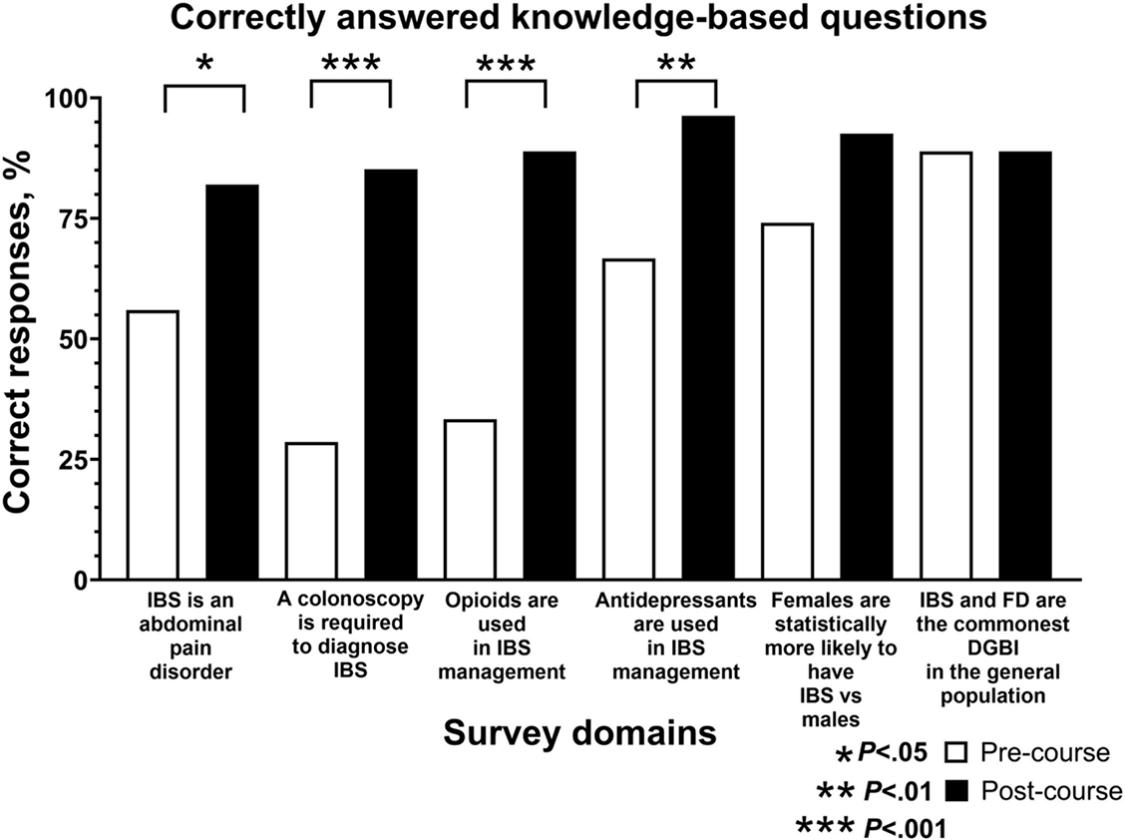
The proportion of correctly answered questions in the knowledge‐based section of the pre‐ and post‐course surveys. DGBI, disorder of gut‐brain interaction; FD, functional dyspepsia; IBS, irritable bowel syndrome. *Disorder of gut‐brain interaction* is the new accepted terminology for *functional gastrointestinal disorder.*

When the responses to the six knowledge‐based questions addressing IBS were summarised and collectively analysed, there was an increase in the percentage of correct responses in the post‐ versus pre‐course survey (88.9% vs. 57.5%, *p* < 0.001). Students' scores increased in 5 of 6 (83.3%) knowledge‐based questions (Figure 1).

### 4.3 Confidence

**FIGURE 2:**
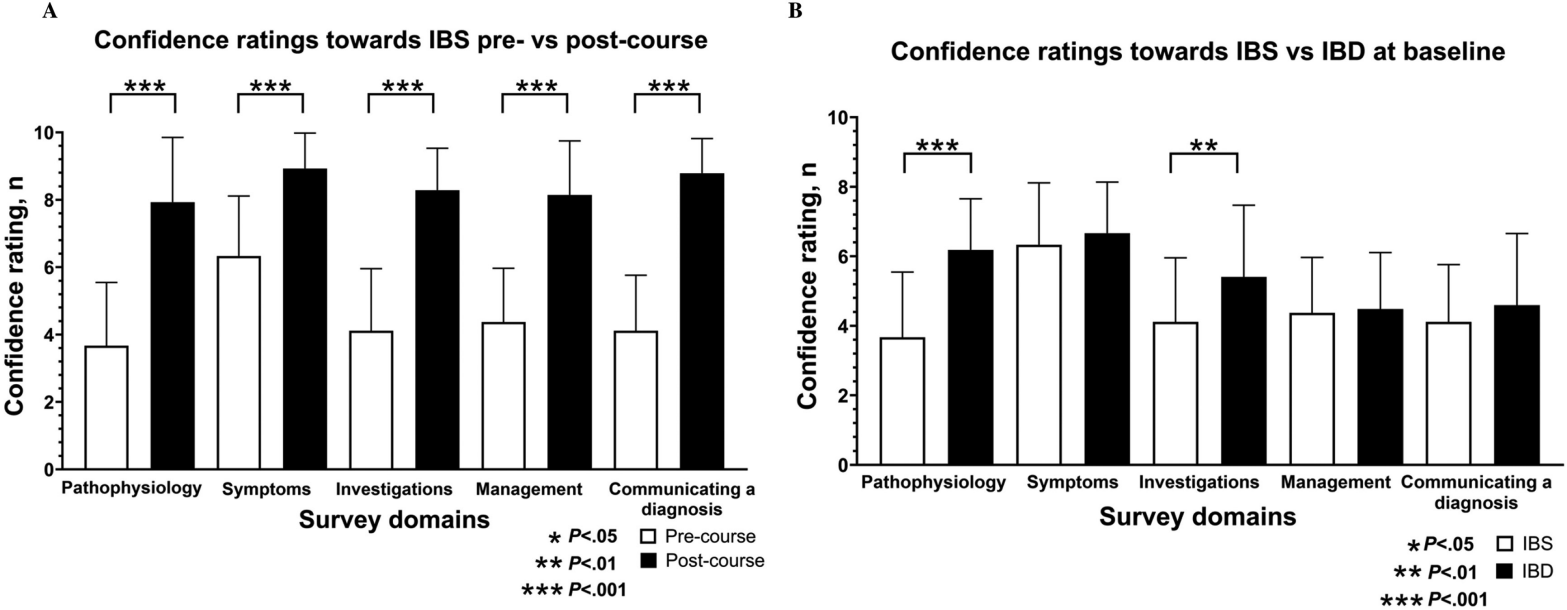
(A) Confidence in domains relating to IBS pre‐ versus post‐course and (B) IBS versus IBD at baseline. IBD, inflammatory bowel disease; IBS, irritable bowel syndrome.

Compared with the pre‐course assessment, students' confidence ratings increased in all domains relating to IBS in the post‐course assessment (Figure 2a). At baseline, participants reported significantly higher confidence ratings around IBD versus IBS (Figure 2b).

### 4.4 Attitudes

Compared with the pre‐course evaluation, there was a statistically significant improvement in attitude ratings in 11 of 12 questions related to IBS (Table 2). Participants expressed less favourable attitudes towards patients with IBS versus IBD in 12 of 12 (100%) questions at baseline, amongst which 10 reached statistical significance (Table 3).

**TABLE 2:** Change in attitudes towards IBS post‐ versus pre‐course.

Question	Pre‐course, mean [SD]	Post‐course, mean [SD]	*p*
Patients with IBS are amenable to referrals	5.70 [1.46]	6.96 [2.03]^a^	0.03
IBS symptoms are exaggerated	3.04 [1.56]	2.32 [1.89]^a^	0.1
Patients with IBS are sick	5.78 [1.40]	7.61 [1.71]^a^	0.001
IBS is a real condition	9.30 [1.20]	9.57 [0.84]^a^	0.3
Patients with IBS are responsible for their condition	1.89 [1.15]	2.43 [2.41]	0.5
Patients with IBS will adhere to the treatment plan	6.00 [1.92]	7.00 [1.87]^a^	0.03
I want to be the healthcare provider for a patient with IBS	6.96 [2.28]	8.43 [2.89]^a^	0.05
It is likely that IBS patients work collaboratively with their healthcare provider	6.78 [1.65]	8.39 [2.71]^a^	0.02
It is likely that IBS patients will thank their provider	6.30 [2.22]	8.29 [2.73]^a^	0.006
Patients with IBS are easy to get along with	6.41 [1.62]	8.04 [2.85]^a^	0.02
Patients with IBS will agree to the treatment plan	5.70 [1.75]	7.18 [1.39]^a^	0.005
Patients with IBS have reasonable expectations	5.89 [1.87]	6.93 [2.05]^a^	0.04

Abbreviation: IBS, irritable bowel syndrome.

^a^ Favourable direction of change on a Likert scale.

**TABLE 3:** Difference in attitudes towards patients with IBS versus IBD pre‐course.

Questions	IBS, mean [SD]	IBD, mean [SD]	*p*
Patients are amenable to referrals	5.70 [1.46]^a^	7.56 [1.74]	< 0.001
Symptoms are exaggerated	3.04 [1.56]^a^	2.04 [1.13]	0.004
Patients are sick	5.78 [1.40]^a^	7.59 [1.28]	< 0.001
The condition is real	9.30 [1.20]^a^	9.70 [0.61]	0.05
Patients are responsible for their condition	1.89 [1.15]^a^	1.48 [0.85]	0.02
Patients will adhere to treatment plan	6.00 [1.92]^a^	7.67 [1.14]	< 0.001
I want to be the healthcare provider for this patient	6.96 [2.28]^a^	7.52 [2.12]	0.16
Patients are likely to work collaboratively with their healthcare provider	6.78 [1.65]^a^	7.67 [1.39]	0.003
Patients are likely to thank their healthcare provider	6.30 [2.22]^a^	7.33 [1.82]	0.004
Patients are easy to get along with	6.41 [1.62]^a^	7.30 [1.56]	< 0.001
Patients will agree to the treatment plan	5.70 [1.75]^a^	7.63 [1.31]	< 0.001
Patients will have reasonable expectations	5.89 [1.87]^a^	6.93 [1.62]	0.01

Abbreviations: IBD; inflammatory bowel disease; IBS, irritable bowel syndrome.

^a^ Unfavourable attitudes towards patients with IBS versus IBD on a Likert scale.

### 4.5 Burnout

Students' mean burnout in the pre‐course questionnaire was 4.11 [1.57], and the mean attitude rating towards patients with IBS was 5.74 [1.44], on Likert scales that reflected higher burnout and increasingly positive attitudes from 1 to 10, respectively. A Spearman rank correlation coefficient between both scores was −0.10 (*p* = 0.6), which suggested no significant correlation between the variables.

### 4.6 Objective Structured Clinical Examination and General Post‐Course Feedback

**FIGURE 3:**
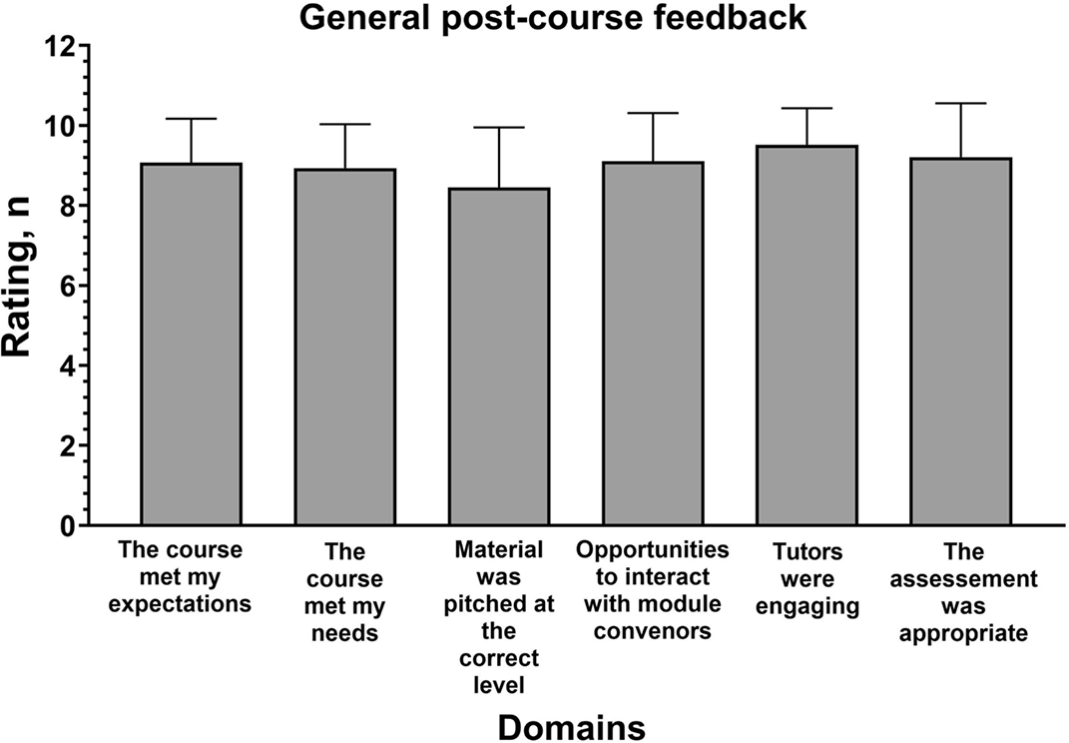
General post‐course feedback. A higher mean score indicates more favourable feedback.

One hundred percent of students passed the OSCE—further information on the OSCE cannot be provided as this may jeopardise the assessment for future years. On a Likert scale (1 to 10) where a higher score represented a more favourable response, the mean scores for all eight questions were consistently above eight (Figure 3). The mean [SD] score for the overall experience was 9.21 [1.13]. Video feedback was uploaded online: https://vimeo.com/952904314?share=copy.

## 5 Implications

An inter‐specialty curriculum is important for teaching related to FMDs, due to the frequent symptom overlap between different organ systems and the need for multidisciplinary management. Consistent with previous work [11], students in our study had less favourable attitudes towards patients with IBS versus IBD. Poorer attitudes towards FMDs versus organic diseases may be propagated through the ‘hidden’ medical curriculum, which refers to the implicit and unscripted messages about values, norms and attitudes, which can have a powerful influence on students' professional development [12]. Stigma, which broadly refers to a social devaluation based on negative stereotypes against a particular population, may be inherited by students from tutors [7]. Educational interventions may help combat stigma and should be implemented during the formative years of clinical training, as we have done in this module. Indeed, at this stage, students are less likely to have inherited negative attitudes from colleagues or tutors through the ‘hidden’ curriculum.

*Educational interventions may help combat stigma and should be implemented during the formative years of clinical training, as we have done in this module*.

Students' confidence ratings around IBS were lower than IBD in domains related to pathophysiology, investigations and communicating a diagnosis, consistent with previous literature comparing confidence around FMDs versus organic diseases [13]. This relatively lower confidence may be partly attributed to the absence of disease biomarkers for diagnosing FMDs, which creates diagnostic uncertainty, the limited availability of evidence‐based treatments and the perceived lack of control in managing these conditions [8].

While burnout has been linked to self‐reported negative attitudes and behaviours towards patients [14], our study did not uncover this association amongst medical students. Experiential teaching has been shown to result in long‐term improvements in students' abilities to take a clinical history from simulated patients—lay persons or actors who adopt and adapt a patient scenario [10]—with chronic pain [15]. We integrated experiential teaching into our curriculum by employing small group teaching and sessions with simulated patients. Training with simulated patients may help students fashion explanations that are acceptable to patients when diagnosing and managing FMDs. Simulated patients were also called upon during the final assessment (OSCE), which was passed by 100% of students.

Key strengths of this curriculum relate to its inter‐disciplinary nature, the variety of teaching methods used (didactic teaching, small group teaching and sessions with simulated patients), the use of surveys in the evaluation that were based on validated questionnaires [11] and an OSCE that adhered to the standards set by the UK Medical Schools Council. Limitations of the study relate to its sample size (*n* = 29) and survey items which were oriented around IBS (based on the condition's high population prevalence and the joint clinical experience of the module convenors), as opposed to the spectrum of FMDs, which limits the generalisability of our findings. Moreover, pre‐ and post‐course responses were analysed per question to identify deficits and where additional instruction may have been needed, but this could have increased the risk of Type I error. Finally, the absence of experimental and control groups, combined with the use of self‐reported questionnaires at a six‐week interval, may have introduced response bias and limited the internal validity of the findings.

*Key strengths of this curriculum relate to its inter‐disciplinary nature, the variety ofteaching methods used (didactic teaching, small group teaching, and sessions with simulatedpatients), the use of surveys in the evaluation that were based on validated questionnaires, and an OSCE that adhered to the standards set by the UK Medical Schools Council.*

Curriculum delivery may have been enhanced had we integrated Balint groups within the course, which foster self‐awareness, empathy and resilience for healthcare professionals who manage patients with chronic health conditions. There is also a need to research whether the improvement in knowledge, attitudes and confidence afforded by this curriculum is durable over the long term and, importantly, in clinical practice.

In conclusion, this inter‐disciplinary curriculum offers clinical educators a template around which to develop a programme of activities to increase knowledge of, confidence around, and attitudes towards patients with FMDs.

## Author Contributions

**Mohsin F. Butt:** conceptualization, methodology, formal analysis, data curation, writing – original draft, writing – review and editing, project administration, visualization, investigation, resources. **Daniele Scotto:** methodology. **Christopher G.S. Gilmartin:** methodology. **Arkadeep Dhali:** formal analysis. **Sherif Gonem:** methodology. **Sumeet Singhal:** methodology. **Tanya M. Monaghan:** conceptualization, methodology, writing – review and editing. **John Frain:** conceptualization, methodology, writing – review and editing. **Maura Corsetti:** conceptualization, methodology, investigation, supervision, writing – review and editing, funding acquisition, resources, data curation, visualization, project administration.

## Conflicts of Interest

The authors declare no conflicts of interest.

## Supporting information


**Data S1.** Supporting information.

**Data S2.** Supporting information.

**Data S3.** Supporting information.

## Data Availability

The data that support the findings of this study are available from the corresponding author upon reasonable request.
