# Supplementary material for: A Medical Student Curriculum on Functional Medical Disorders
Source: Clin Teach. 2025 Jun 26;22(4):e70117. doi: 10.1111/tct.70117 (PMC12202073; doi:10.1111/tct.70117)
Supplement: Supplementary file 1 — Data S1. Supporting information. [file TCT-22-e70117-s001.pdf]

Title:
Assessing medical students' knowledge of, confidence around, and attitudes towards functional somatic disorders pre- and post-module

Survey lead:
Mohsin Butt, B.Sc.(Hons) M.B.B.S.(London)

Contact person for all queries related to this survey:
Dr Mohsin Butt
NIHR Academic Clinical Fellow in Gastroenterology, Neurogastroenterology Unit, University of Nottingham
mohsin.butt@nottingham.ac.uk

University of Nottingham Ethics Approval Number:
FMHS 44-1023

You are being asked to voluntarily complete this survey as you are enrolled on the BMedSci module for medical students titled *Introduction to Functional Somatic Disorders.* The questions in this survey relate to your awareness of functional somatic disorders and your attitudes towards patients with these conditions. You will be asked to complete surveys prior to enrolling on the course and following course completion.

Completing this survey is completely **voluntary**. The results of this survey will **not** impact the final grade that you will receive. By agreeing to complete this survey you are also consenting to the (anonymous) data being published in a peer-reviewed journal and being presented at scientific symposia. Any publications that use the data from this survey will **not** include your personal information.

This survey will take approximately 15 minutes to complete, and a post-course survey will be sent to you following the completion of this module.

You can choose not to answer any question. You can also choose to stop taking the survey at any time.

The possible risks to you of completing this survey are:

- Being exposed to questions that may cause a strong emotional reaction if you or someone you know has experience of living with a functional medical disorder
- Having someone else find out that you were in a research study
- Potential loss of confidentiality of data

The potential benefits to you of completing this survey are:

- Reflecting on your knowledge and experience of functional medical disorders
- Helping to improve this course in subsequent academic years

BACKGROUND INFORMATION

1. **What is your sex?**
    a. Female
    b. Male
    c. Other
    d. Prefer not to say

2. **What is your age? ……….**

3. **Have you studied another degree prior to entering medical school?**
    a. Yes
    b. No
    c. Prefer not to say

4. **Has neurogastroenterology and motility been addressed in your (taught) medical school curriculum to-date?**
    a. Yes
    b. No
    c. Don’t know

5. **Have you received any extra-curricular opportunities (e.g., clinics / electives / informal teaching) in neurogastroenterology and motility?**
    a. Yes
    b. No
    c. Don’t know

TRUE/FALSE QUESTIONS RELATED TO FUNCTIONAL GASTROINTESTINAL DISORDERS

1. **True/false: Irritable bowel syndrome (IBS) is an abdominal pain disorder.**
    a. True
    b. False
    c. Don’t know

2. **True/false: people who meet the diagnostic criteria for IBS must undergo a colonoscopy to confirm the absence of a structural problem before receiving an IBS diagnosis.**
    a. True
    b. False
    c. Don’t know

3. **True/false: Opioid-based medications (such as codeine and morphine) have been proven to be effective in the management of pain related to IBS.**
    a. True
    b. False
    c. Don’t know

4. **True/false: Antidepressants (tricyclic antidepressants and selective serotonin reuptake inhibitors) have been proven to be effective in the management of pain related to IBS.**
    a. True
    b. False
    c. Don't know

5. **True/false: Females are statistically more likely than males to develop IBS.**
    a. True
    b. False
    c. Don't know

6. **True/false: IBS and functional dyspepsia are the commonest functional gastrointestinal conditions in the UK general population.**
    a. True
    b. False
    c. Don't know

FAMILIARITY WITH IRRITABLE BOWEL SYNDROME

PATHOPHYSIOLOGY

1. **How familiar are you with the underlying pathophysiological mechanism of ulcerative colitis?**

1 2 3 4 5 6 7 8 9 10
Very unfamiliar **Very familiar**

2. **How familiar are you with the underlying pathophysiological mechanism of irritable bowel syndrome?**

1 2 3 4 5 6 7 8 9 10
Very unfamiliar Very familiar

CLINICAL SYMPTOMS

3. **How familiar are you with the clinical symptoms of ulcerative colitis?**

1 2 3 4 5 6 7 8 9 10
Very unfamiliar Very familiar

4. **How familiar are you with the clinical symptoms of irritable bowel syndrome?**

1 2 3 4 5 6 7 8 9 10
Very unfamiliar Very familiar

INVESTIGATIONS

5. **How familiar are you with the investigations that should be ordered to diagnose ulcerative colitis?**

1 2 3 4 5 6 7 8 9 10
Very unfamiliar Very familiar

6. **How familiar are you with the investigations that should be ordered to diagnose irritable bowel syndrome?**

1 2 3 4 5 6 7 8 9 10
Very unfamiliar Very familiar

MANAGEMENT

7. **How familiar are you with the management of ulcerative colitis?**

1 2 3 4 5 6 7 8 9 10
Very unfamiliar Very familiar

8. **How familiar are you with the management of irritable bowel syndrome?**

1 2 3 4 5 6 7 8 9 10
Very unfamiliar Very familiar

COMMUNICATION

9. **How confident do you feel about communicating an ulcerative colitis diagnosis to patients?**

1 2 3 4 5 6 7 8 9 10
Very unfamiliar Very familiar

10. **How confident do you feel about communicating an irritable bowel syndrome diagnosis to patients?**

1 2 3 4 5 6 7 8 9 10

Very unfamiliar Very familiar

PATIENT VIGNETTES

Patient A (pronouns: they/them) was referred to an outpatient gastroenterology clinic with a one-year history of central abdominal pain which affected them daily. The abdominal pain was relieved by emptying their bowels and their stools were often watery in consistency. They emptied their bowels up to four times a day. They also reported feeling very full-up shortly after eating food and experienced an unpleasant sensation (akin to the persistence of food) in the upper part of the stomach after eating (known as post-prandial fullness). They had a past medical history of anorexia nervosa, depression, and fibromyalgia. They underwent a comprehensive work-up recommended by UK NICE guidelines and were diagnosed with irritable bowel syndrome and functional dyspepsia. They were referred to a dietitian to help identify trigger foods and a psychologist to receive cognitive behavioural therapy.

1. **How likely do you think it is that this patient will be amenable to the above referrals?**

   1 2 3 4 5 6 7 8 9 10
   Highly unlikely Highly likely

2. **To what extent do you feel that this patient's symptoms may be exaggerated?**

   1 2 3 4 5 6 7 8 9 10
   Not at all Completely

3. **How sick do you think this patient is?**

   1 2 3 4 5 6 7 8 9 10
   Not at all Completely

4. **Do you think that this is a 'real' illness?**

   1 2 3 4 5 6 7 8 9 10
   Not at all Completely

5. **To what degree do you feel that this patient is responsible for their illness?**

   1 2 3 4 5 6 7 8 9 10
   Not at all Completely

6. **Do you think that this patient would be able to successfully adhere to the treatment plan?**

   1 2 3 4 5 6 7 8 9 10
   Not at all Completely

7. **How much would you want to be a healthcare provider for this patient?**

   1 2 3 4 5 6 7 8 9 10
   Not at all Completely

8. **How likely do you feel that this patient would be able to work collaboratively with you in the treatment process?**

   1 2 3 4 5 6 7 8 9 10
   Highly unlikely Highly likely

9. **How likely do you feel that this patient will thank you for your guidance?**

   1 2 3 4 5 6 7 8 9 10
   Highly unlikely Highly likely

10. **To what extent do you feel that this patient may be easy to get along with?**

    1 2 3 4 5 6 7 8 9 10
    Not at all Completely

11. **How likely do you feel that this patient will agree with your treatment plan?**

    1 2 3 4 5 6 7 8 9 10
    Highly unlikely Highly likely

12. **How likely do you think it is that this patient will have reasonable expectations?**

    1 2 3 4 5 6 7 8 9 10
    Highly unlikely Highly likely

How likely do you feel it is for this patient to demonstrate each of the following, relative to their condition?

13. **Patience**

    1 2 3 4 5 6 7 8 9 10
    Highly unlikely Highly likely

14. **Optimism**

    1 2 3 4 5 6 7 8 9 10
    Highly unlikely Highly likely

15. **Resilience**

1 2 3 4 5 6 7 8 9 10

Highly unlikely Highly likely

16. Honesty

1 2 3 4 5 6 7 8 9 10

Highly unlikely Highly likely

Patient B (pronouns: they/them) was referred to an outpatient gastroenterology clinic with a one-year history of worsening episodic right lower quadrant abdominal pain associated with loose, bloody, mucus-covered stools. They described an urgency to evacuate their bowels which frequently awoke them from sleep. They had a past medical history of pernicious anaemia. They underwent a comprehensive work-up recommended by UK NICE guidelines and were diagnosed with Crohn's disease (a form of inflammatory bowel disease). Alongside drug therapy, they were referred to a dietitian to help manage nutrient deficiencies and a psychologist to help them manage their associated psychological co-morbidity.

1. **How likely do you think it is that this patient will be amenable to the above referrals?**

   1 2 3 4 5 6 7 8 9 10
   Highly unlikely Highly likely

2. **To what extent do you feel that this patient's symptoms may be exaggerated?**

   1 2 3 4 5 6 7 8 9 10
   Not at all Completely

3. **How sick do you think this patient is?**

   1 2 3 4 5 6 7 8 9 10
   Not at all Completely

4. **Do you think that this is a 'real' illness?**

   1 2 3 4 5 6 7 8 9 10
   Not at all Completely

5. **To what degree do you feel that this patient is responsible for their illness?**

   1 2 3 4 5 6 7 8 9 10
   Not at all Completely

6. **Do you think that this patient would be able to successfully adhere to the treatment plan?**

   1 2 3 4 5 6 7 8 9 10
   Not at all Completely

7. **How much would you want to be a provider for this patient?**

   1 2 3 4 5 6 7 8 9 10

Not at all Completely

8. **How likely do you feel that this patient would be able to work collaboratively with you in the treatment process?**

1 2 3 4 5 6 7 8 9 10
Highly unlikely Highly likely

9. **How likely do you feel that this patient will thank you for your guidance?**

1 2 3 4 5 6 7 8 9 10
Highly unlikely Highly likely

10. **To what extent do you feel that this patient may be easy to get along with?**

1 2 3 4 5 6 7 8 9 10
Not at all Completely

11. **How likely do you feel that this patient will agree with your treatment plan?**

1 2 3 4 5 6 7 8 9 10
Highly unlikely Highly likely

12. **How likely do you think it is that this patient will have reasonable expectations?**

1 2 3 4 5 6 7 8 9 10
Highly unlikely Highly likely

How likely do you feel it is for this patient to demonstrate each of the following, relative to their condition?

13. **Patience**

1 2 3 4 5 6 7 8 9 10
Highly unlikely Highly likely

14. **Optimism**

1 2 3 4 5 6 7 8 9 10
Highly unlikely Highly likely

15. **Resilience**

1 2 3 4 5 6 7 8 9 10
Highly unlikely Highly likely

16. Honesty

1 2 3 4 5 6 7 8 9 10
Highly unlikely Highly likely

At the end of the survey, please reflect on the clinical vignettes you read about and answer the following questions.

1. **I feel burned out from medical school**

   1 2 3 4 5 6 7 8 9 10
   Not at all Completely

2. **I have become more callous toward people since starting medical school**

   1 2 3 4 5 6 7 8 9 10
   Not at all Completely

3. **What was most surprising to you when completing this survey? (free text)**
