# Supplementary material for: A Medical Student Curriculum on Functional Medical Disorders
Source: Clin Teach. 2025 Jun 26;22(4):e70117. doi: 10.1111/tct.70117 (PMC12202073; doi:10.1111/tct.70117)
Supplement: Supplementary file 2 — Data S2. Supporting information. [file TCT-22-e70117-s003.pdf]

To what extent do you agree or disagree with the following statements:

1. **The course met my expectations**

1 2 3 4 5 6 7 8 9 10
Strongly disagree Strongly agree

2. **The course met my needs**

1 2 3 4 5 6 7 8 9 10
Strongly disagree Strongly agree

3. **The information was pitched at the right level, e.g. too low/simple, too high/complex**

1 2 3 4 5 6 7 8 9 10
Strongly disagree Strongly agree

4. **I was provided with many opportunities to interact with the module convenors**

1 2 3 4 5 6 7 8 9 10
Strongly disagree Strongly agree

5. **The module convenors were engaging**

1 2 3 4 5 6 7 8 9 10
Strongly disagree Strongly agree

6. **The assessment used was appropriate**

1 2 3 4 5 6 7 8 9 10
Strongly disagree Strongly agree

PLEASE TURN OVER

7. Rate your overall experience of this course

1 2 3 4 5 6 7 8 9 10
Very poor Very good

8. How likely are you to recommend this course to a friend or colleague?

1 2 3 4 5 6 7 8 9 10
Very unlikely Very likely

9. What will you do differently now you've attended the course? (free text)

10. Any other feedback? (free text)
