# Supplementary material for: A Medical Student Curriculum on Functional Medical Disorders
Source: Clin Teach. 2025 Jun 26;22(4):e70117. doi: 10.1111/tct.70117 (PMC12202073; doi:10.1111/tct.70117)
Supplement: Supplementary file 3 — Data S3. Supporting information. [file TCT-22-e70117-s002.pdf]

PROFESSOR MAURA CORSETTI AND DR MOHSIN F. BUTT

NIHR Nottingham BRC, Nottingham University Hospitals NHS Trust and the University of Nottingham, Nottingham, UK. Nottingham Digestive Diseases Centre, Translational Medical Sciences, School of Medicine, University of Nottingham, Nottingham, UK.

Photograph, Video or Audio Recording Consent Form

RE: VIDEO RECORDING FOR THE *INTRODUCTION TO FUNCTIONAL MEDICAL DISORDERS MODULE*

I, ____ (recorded person's full name), do hereby consent to the use by **PROFESSOR MAURA CORSETTI AND DR MOHSIN F. BUTT** of my image, video, voice, or all three of them, in the item described above.

In addition, I waive any right to inspect or approve the finished video recording.

I agree that all such pictures, video or audio recordings and any reproduction thereof shall remain the property of **PROFESSOR MAURA CORSETTI AND DR MOHSIN F. BUTT** and both individuals may use it as it they fit.

I understand that this consent is perpetual, that I may not revoke it, and that it is binding.

I understand that this video may appear publicly.

Name: ____

Date of Birth: _____ / _____/ __________

Signature:____

Date: _____ / _____ / __________
